# Rapid dehydration of grape berries dampens the post-ripening transcriptomic program and the metabolite profile evolution

**DOI:** 10.1038/s41438-020-00362-5

**Published:** 2020-09-01

**Authors:** Sara Zenoni, Alessandra Amato, Erica D’Incà, Flavia Guzzo, Giovanni Battista Tornielli

**Affiliations:** Department of Biotechnology, University of Verona, Strada Le Grazie 15, 37134 Verona, Italy

**Keywords:** Secondary metabolism, Gene expression

## Abstract

The postharvest dehydration of grape berries allows the concentration of sugars and other solutes and promotes the synthesis of metabolites and aroma compounds unique to high-quality raisin wines such as the passito wines made in Italy. These dynamic changes are dependent on environmental parameters such as temperature and relative humidity, as well as endogenous factors such as berry morphology and genotype, but the contribution of each variable is not well understood. Here, we compared berries subjected to natural or accelerated dehydration, the latter driven by forced air flow. We followed the evolution of transcript and metabolite profiles and found that accelerated dehydration clearly dampened the natural transcriptomic and metabolomic programs of postharvest berries. We found that slow dehydration over a prolonged duration is necessary to induce gene expression and metabolite accumulation associated with the final quality traits of dehydrated berries. The accumulation of key metabolites (particularly stilbenoids) during postharvest dehydration is inhibited by rapid dehydration conditions that shorten the berry life time.

## Introduction

Most of the physical and chemical quality traits sought by grape growers and winemakers are acquired by the berries during ripening, which for wine grapes may last 30–60 days or more, depending on the cultivar, agronomic/environmental factors, and winemaker choice. The main features of berry ripening include softening, an increase in size, the accumulation of sugars, the loss of acidity, the accumulation of anthocyanin (in red berry cultivars) and aroma compounds^1^. For enological purposes, berry life can be extended by placing harvested grapes in an environment that prolongs cell survival and metabolic activity. This practice is applied during the production of premium passito wines such as Amarone and Recioto, and it involves the progressive partial dehydration of berries, which increases the concentration of sugars and other solutes, and encourages the accumulation of unique aroma compounds^2^. The dynamic changes that occur during this process are dependent on environmental parameters and endogenous factors, such as berry morphology and genotype^3,4^. In agreement with basic dehydration theory, the rate of dehydration increases under conditions of high temperature and low relative humidity, although temperature has a much greater impact than humidity on berry cell metabolism^4^.

Genome-wide gene expression analysis has been used to study grape berry development and has provided a comprehensive and detailed overview of the molecular program controlling the physical and biochemical changes during berry formation and ripening. The application of such large-scale analytical methods to postharvest life has revealed that a new transcriptomic program emerges following the detachment of grape clusters from the vine, ultimately driving the metabolic changes that determine the final quality traits of dehydrated berries^5–7^. The key features of this transcriptomic program include the massive induction of genes controlling stilbenoid and terpenoid biosynthesis, the oxidative polymerization of phenol compounds, and the modification of the cell wall pectin fraction. These processes are induced in part by the osmotic stress caused by progressive water loss^8^.

Different grape varieties subjected to postharvest dehydration under identical controlled conditions differ in terms of dehydration kinetics, probably reflecting their distinct morpho-anatomical properties, such as berry size, skin thickness, and cuticle features. Varieties with a slow dehydration rate take longer to achieve the desired weight loss, and generally show more extreme modulation of gene expression^7^. This indicates that the rate of dehydration and the duration of the dehydration period may influence the transcriptomic program within any limits set by the genetic background of each variety^9^. Previous investigations provided evidence for a positive correlation between the slow rate of dehydration and the intensity of the transcriptional response in berry skin by comparing a very short and fast dehydration process to a much longer and slower one^10,11^. However, the relationship between genome-wide gene expression and the dehydration rate, weight loss and duration of dehydration in grape berries has not been evaluated in detail.

We therefore designed an experimental strategy to compare berries of a single genotype collected at the same weight loss intervals or at the same times after harvest during two dehydration processes, differing only in the dehydration rate. Grapes dried under natural conditions were compared with those dried by forced air flow, nearly doubling the rate of dehydration by removing the moisture from the berry microenvironment. We found that increasing the dehydration rate dampens the transcriptomic changes and the accumulation of metabolites, confirming that the rate and duration of dehydration are key parameters affecting the post-ripening transcriptomic program and thus the final quality traits of grape berries.

## Results

### Postharvest dehydration under two different environmental conditions

**Fig. 1:**
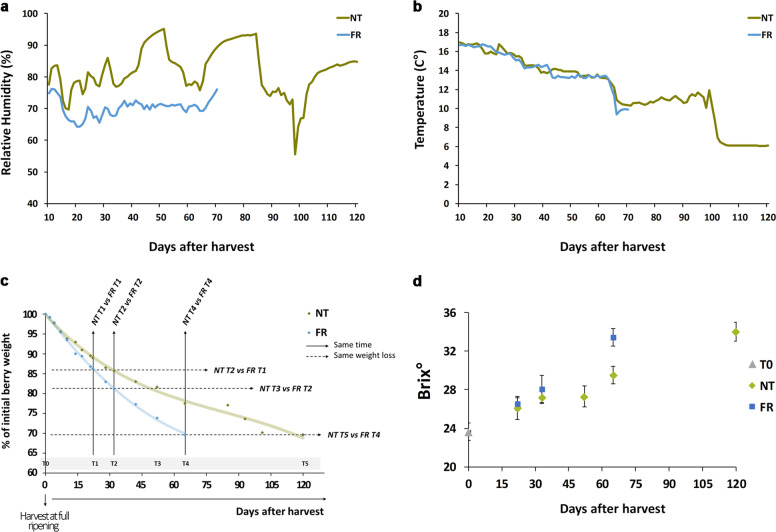
Overview of the two postharvest dehydration processes and sampling strategy. **a** Relative humidity of the two dehydrating rooms used to reproduce natural (NT) and forced (FR) dehydration processes. **b** Temperature of the two dehydrating rooms. **c** Dehydration kinetics of berries under NT and FR conditions. The six sampling time points for berries under NT conditions and the four sampling time points for berries under FR conditions are shown together with the indication of samples collected at the same time or at the same level of weight loss. **d** Total soluble solids concentration during the NT and FR processes. Error bars represent standard deviations (*n* = 3)

Corvina berries (vintage 2011) were harvested at commercial maturity (23.6 ± 0.9 °Brix). The detached bunches were then randomly assigned to two groups and stored on wooden trays in different rooms for dehydration, with thermo-hygrometric conditions recorded by probes placed very close to the bunches. The first room provided a natural environment (NT), defined as postharvest dehydration conditions typical of the Verona region (temperature gradually declining from 16 °C to 7 °C and relative humidity generally >75%, with wide variations). In the second room, bunches were subjected to intense ventilation by forced air (FR), significantly reducing the relative humidity compared with the first room, without changes in temperature (Fig. 1a, b). The dehydration process was stopped when the berries had lost 30% of their initial weight. Under NT conditions, the dehydration process took ~120 days, whereas under FR conditions the same weight loss occurred after ~60 days (Fig. 1c; Table S1). We also monitored the total soluble solids concentration and the berry juice acidity.

As expected, the soluble solids concentration increased more rapidly under FR conditions, and similar °Brix values were achieved at the end of both processes (Fig. 1d). In contrast, similar titratable acidity and pH values were recorded during both processes at the same time points (Table S2). Variation in titratable acidity reflects the cumulative effect of juice concentration due to water loss and the depletion of malic acid owing to respiration^12^, leading to final values similar to those observed at harvest.

In order to dissect the effects of water loss stress and the time after harvest on berry gene expression and metabolism, berries were sampled during dehydration to allow comparisons between the NT and FR conditions based on the same time after harvest or the same percentage weight loss (Fig. 1c). Accordingly, NT berries were collected at six time points (T0, T1, T2, T3, T4, and T5), whereas FR berries were collected at four time points (T0, T1, T2, and T4). For both conditions, T0 represented fully ripe berries, and T1, T2, and T4 were 22, 33, and 65 days after harvest, respectively. For the NT berries, T3 and T5 were 52 and 120 days after harvest, respectively. In terms of weight loss, NT-T2 and FR-T1 corresponded to 14% weight loss, NT-T3 and FR-T2 corresponded to 18% weight loss, and NT-T5 and FR-T4 corresponded to 30% weight loss (Fig. 1c).

### Metabolomic changes in grape berries during postharvest dehydration

**Fig. 2:**
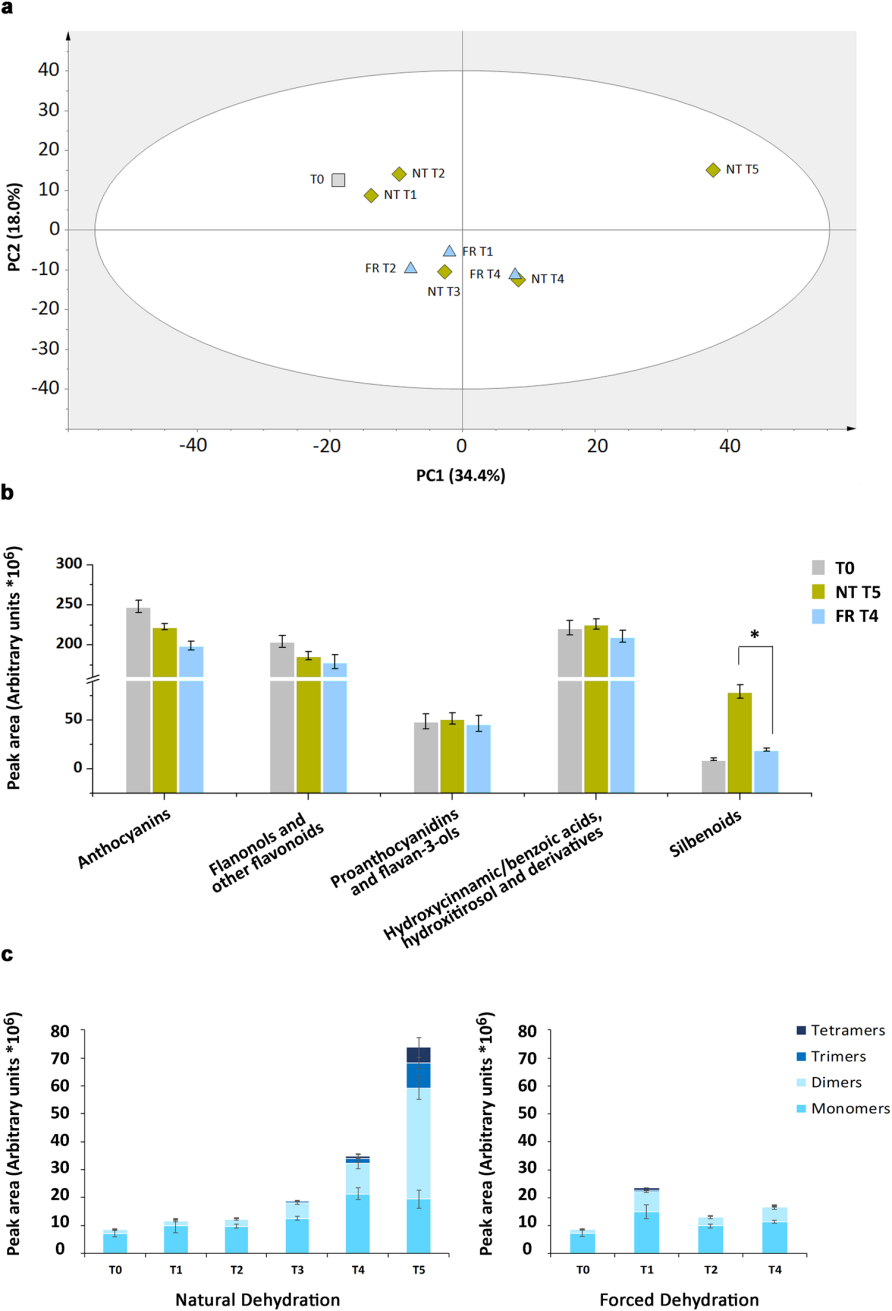
The dynamic berry metabolome under NT and FR postharvest dehydration conditions. **a** PCA score scatter plot of non-volatile metabolites from Corvina berries collected during postharvest dehydration under NT and FR conditions. The PCA score scatter plot is colored according to the condition. **b** Distribution of the five classes of phenylpropanoid-related metabolites at T0, NT-T5, and FR-T4. The distribution is determined by HPLC-MS analysis and is indicated as the sum of the peak areas (arbitrary units) of the molecules belonging to each class (Data S1). Asterisk indicates a significant difference (*t* test, *p* < 0.01). Error bars represent standard deviations. **c** Distribution of stilbenes in berries at all stages of the NT and FR dehydration processes. The details of resveratrol monomers, dimers, trimers, and tetramers are indicated for each time points in different shades of blue

High-performance liquid chromatography with electrospray ionization mass spectrometry (HPLC-ESI-MS) was used to measure the non-volatile metabolites in the Corvina berries collected during postharvest dehydration under the two different conditions. Among the 825 recorded *m/z* features, 235 were putatively assigned to molecules, aglycones, fragments, and molecular adducts, and the remaining 590 remained unidentified. The identified metabolites included 79 anthocyanins, 22 proanthocyanidins, 48 other flavonoids, 22 hydroxycinnamic acids, three hydroxybenzoic acids, four hydroxytyrosols, 46 stilbenes, and a small number of sugars and nonaromatic organic acids (Data S1). The metabolic changes during postharvest dehydration were investigated by normalizing the data matrix to the level of weight loss, in order to minimize the concentration effect. Principal component analysis (PCA) revealed that the FR-T1/FR-T2 berries were clearly separated from the T0 berries and also from the NT-T1/NT-T2 berries, but there was less separation between the T0 and NT-T1/NT-T2 berries (Fig. 2a). The FR-T1/FR-T2 berries grouped with NT-T3, whereas the FR-T4 berries grouped with NT-T4, showing that berries reached a similar metabolic profile after 65 days of postharvest dehydration (T4) although the dynamics of each process were distinct. PCA also revealed a marked change in the NT-T5 berry metabolome, characterized by a very high PC1 score, predominantly caused by the accumulation of stilbenes and the loss of anthocyanins representing the highest and lowest values of the PC1 loadings, respectively (Data S2). The annotated metabolites were then grouped into five classes as follows: anthocyanins (including anthocyanidin-3-*O*-glucosides, acetyl-anthocyanins and coumaroyl-anthocyanins), flavonols and other flavonoids, proanthocyanins and flavan-3-ols, hydroxycinnamic/hydroxybenzoic acids and hydroxityrosol and derivatives, and stilbenoids. The comparison between classes, calculated by summing the total intensities of metabolites at the beginning (T0) and at the end of dehydration (FR-T4 and NT-T5), revealed a significant difference only for the stilbenoids, which accumulated to high levels under NT conditions but only increased marginally in response to forced intense ventilation (Fig. 2b, c).

The degree of stilbene polymerization also differed between two conditions, with a steady increase in dimers, trimers, and tetramers in the NT berries but no significant accumulation of polymeric forms in the FR berries (Fig. 2c). The four other compound classes accumulated to slightly higher levels in NT-T5 compared with FR-T4 berries but the difference was not statistically significant (Fig. 2b).

### Transcriptomic changes in grape berries during postharvest dehydration

**Fig. 3:**
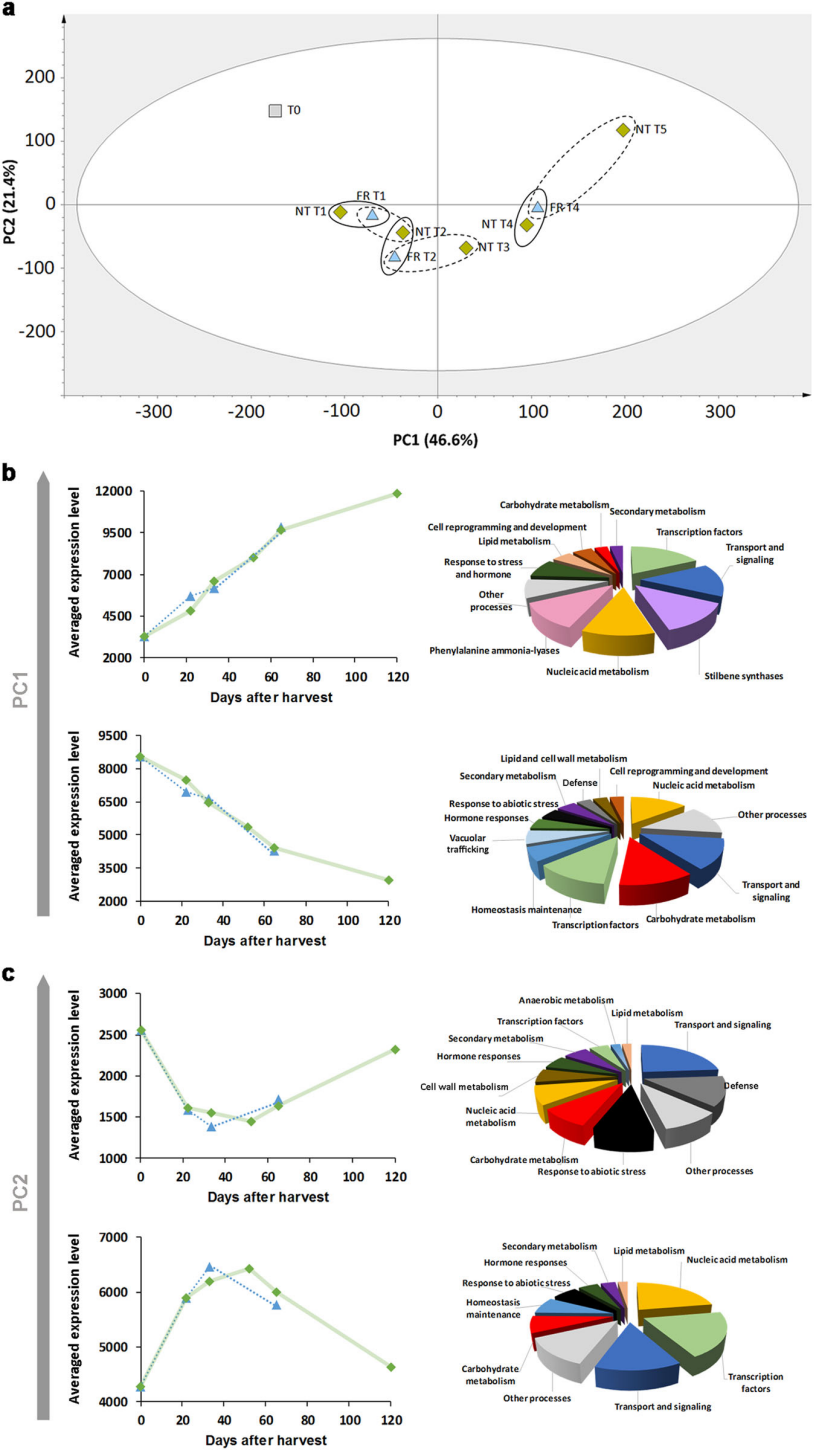
The dynamic berry transcriptome under NT and FR postharvest dehydration conditions. **a** PCA score scatter plot of transcripts detected by microarray analysis in Corvina berries collected during postharvest dehydration under NT and FR conditions. **b** Averaged expression profiles (on the left) and functional category distribution (on the right) of the 100 genes with the highest (on the top) and lowest (on the bottom) PC1 scores. **c** Averaged expression profiles (on the left) and functional category distribution (on the right) of the 100 genes with the highest (on the top) and lowest (on the bottom) PC2 scores

Transcriptomic analysis was carried out on the same samples used for metabolomic analysis (Data S3). PCA applied to the nine berry samples revealed that postharvest transcriptomic changes were primarily described by PC1 and PC2, overall explaining the 68% of total variance (Fig. 3a). PC1 (explaining 46.6% of the variance) clearly reflected the gradual transcriptomic changes occurring throughout the dehydration period, whereas PC2 (explaining 21.4% of the variance) mainly described transient changes in gene expression. The same trends in sample distribution were observed when PCA was used for the reanalysis (Figure S1) of previously reported transcriptomic data representing Corvina berries collected at six postharvest time points^7^.

PCA showed that samples collected at the same time after harvest (solid ovals) were more closely related than samples characterized by the same weight loss (dashed ovals). Indeed, NT-T2 was very close to FR-T2, and NT-T4 very close to FR-T4, whereas a greater “transcriptomic distance” was evident between NT-T3 and FR-T2 and between NT-T5 and FR-T4. Although sample distribution along both principal components mainly reflected the time after harvest, separation along PC2 also revealed that the percentage weight loss played a key role in the induction of common transcriptional changes, at least in samples FR-T2 and NT-T3. These shared the same percentage weight loss (18%) and similar PC2 scores (Fig. 3a).

To identify the most important genes associated with sample distribution, we ranked the 100 genes with the highest and lowest PC1 and PC2 scores (Fig. 3b, c). Focusing on the averaged expression profiles of PC1-related genes, we observed a steady increase in expression levels for genes with the highest (positive) PC1 values and a steady decrease for those with the lowest (negative) PC1 values (Fig. 3b). Differences between the two postharvest dehydration conditions were apparent at T1, where both positive and negative modulation of gene expression was more extreme in FR berries compared with NT berries. The averaged expression level at T4 was the same for both positive and negative PC1-related genes under both dehydration conditions, whereas the averaged expression level was highest for positive PC1 genes and lowest for negative PC1 genes in sample NT-T5 (Fig. 3b). The functional annotation of genes with the highest (positive) PC1 values revealed the induction of many genes encoding stilbene synthases, phenylalanine ammonia-lyases, and stress and hormone response proteins (Fig. 3b, Data S4), in agreement with earlier studies^7^. Genes involved in transport and signaling, nucleic acid metabolism and transcriptional regulation were also strongly induced. The lowest (negative) PC1 scores were associated with genes involved in the ripening phase, including genes involved in carbohydrate, lipid and cell wall metabolism, and hormone responses. These were strongly downregulated during postharvest dehydration (Fig. 3b, Data S4).

Plotting the averaged expression profiles of PC2-related genes revealed the transient downregulation of genes with positive PC2 scores and the transient induction of those with negative PC2 scores (Fig. 3c). These genes, and especially those with positive scores, were generally characterized by lower expression levels than PC1-related genes throughout dehydration. Differences between the two dehydration conditions were observed at T2, with stronger modulation of PC2-related genes in FR compared with NT berries. Furthermore, the averaged expression levels of these genes in the FR-T2 berries appeared to anticipate the expression levels in the NT-T3 berries, suggesting that 18% weight loss is the driving force for this transient modulation of gene expression. As observed for the PC1-related genes, there was a significant difference in the expression of PC2-related genes when comparing samples NT-T5 and FR-T4, which are characterized by the same 30% weight loss.

The functional annotation of the genes with negative PC2 scores (those induced most strongly in the NT-T3 and FR-T2 berries) revealed nucleic acid metabolism, transcription factor activity, and cellular homeostasis as key categories (Fig. 3c, Data S4). The presence of several genes involved in nucleic acid metabolism, including genes encoding six pentatricopeptide (PPR) repeat-containing proteins, a methyl-CpG-binding domain, two DNA replication complexes and several zinc finger domain proteins, strongly suggests that the initial phases of postharvest dehydration are characterized by the profound reorganization of genetic information processing activity in the berry. The functional annotation of genes with the highest PC2 scores (those expressed strongly in T0 and NT-T5 berries, but downregulated in NT-T3 and FR-T2 berries) revealed they encoded proteins involved in abiotic stress responses (such as a dehydration response element-binding transcription factor, a thioredoxin, the glutathione-S-transferase GSTU7, a peroxidase, and a heat shock protein), in defense (such as three disease resistance proteins and three R proteins), and in transport and signaling (such as a jasmonate *O*-methyltransferase and three Ca^2+^-ATPases involved in stress adaptations) (Fig. 3c, Data S4). Two alcohol dehydrogenases and two ERF/AP2 dehydration response element-binding transcription factors also followed the same trend.

Although dehydration may represent a driving force for steady or transient gene modulation, our data suggest that certain stress responses are activated only after a long dehydration period, and this may affect the metabolite profile of berries at the end of the process.

### Dissection of weight loss and time effects on berry transcriptome modulation

**Fig. 4:**
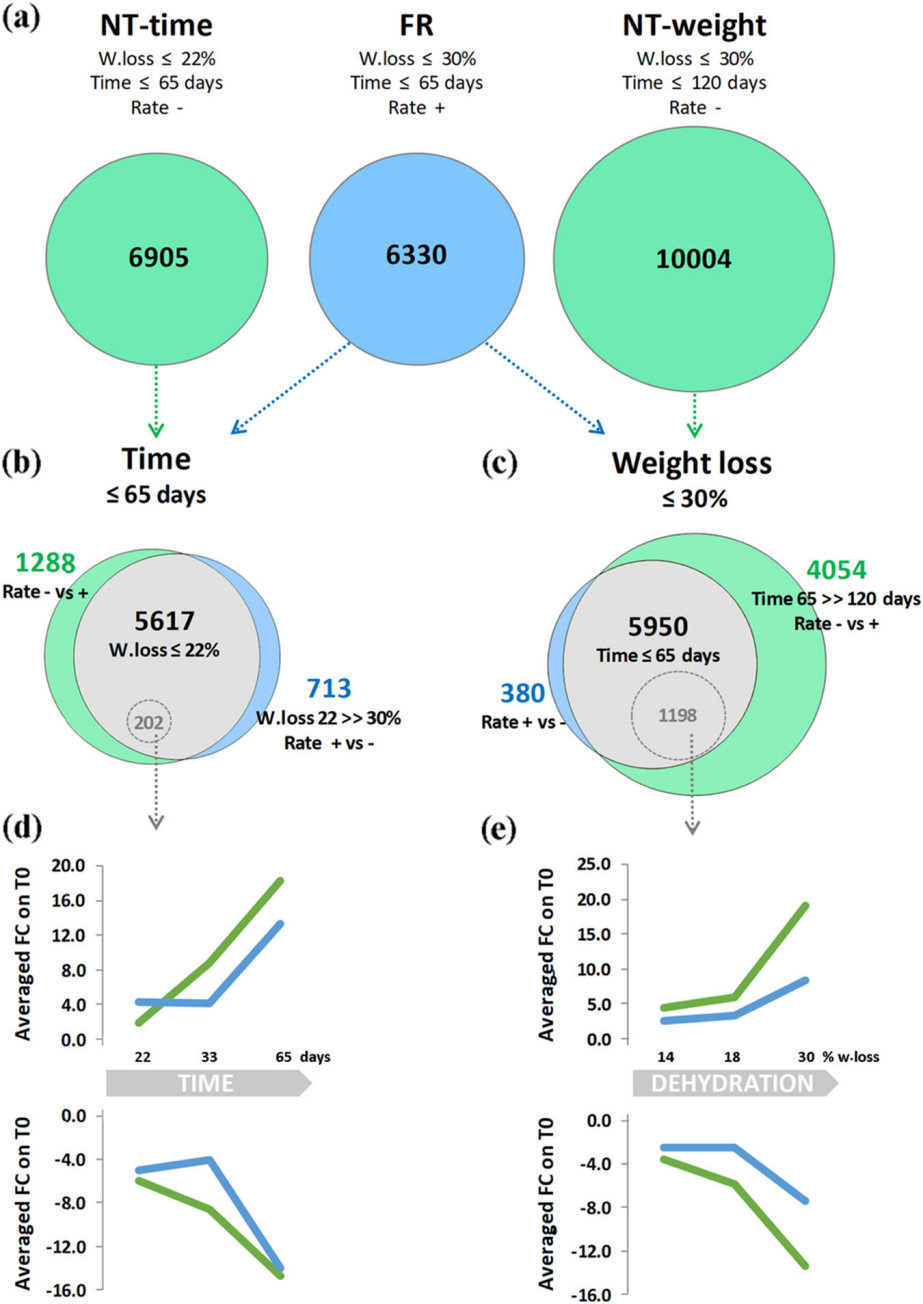
Dissection of weight loss, time, and dehydration rate effects on berry transcriptional changes during postharvest dehydration. **a** Differentially expressed genes (DEGs) of the NT-time data set, NT-weight data set, and FR data set. **b** Venn diagram of common and specific DEGs obtained by comparing the NT-time and FR data set DEGs. **c** Venn diagram of common and specific DEGs obtained by comparing the NT-weight and FR data set DEGs. **d**–**e** Average fold change (FC) relative to T0 for **d** 202 genes with |FC | ≥ 2 between NT-time and FR sample at the same time point in at least one comparison and **e** 1198 genes with |FC | ≥ 2 between NT-weight and FR sample at the same time point in at least one comparison. On the top are reported the expression trends of upregulated genes and on the bottom the trends on the downregulated ones, during dehydration under NT (green) and FR (bleu) conditions. *W loss* weight loss

The transcriptomic PCA distribution revealed a closer relationship between berry samples collected at the same time after harvest than samples with the same degree of weight loss (Fig. 3a). We therefore compared gene modulation during FR and NT dehydration by separating the NT data set into two sample groups: one containing samples collected at same time as the FR samples (T0, NT1, NT2, and NT4; NT-time data set) and the other containing samples with the same weight loss as the FR samples (T0, NT2, NT3, and NT5; NT-weight data set) (Fig. 1c). We then identified differentially expressed genes (DEGs), defined as those with at least a twofold change in expression between T0 and one of the other three time points (|FC | ≥ 2). We identified 6330 genes that were differentially expressed during the FR dehydration process, a similar number (6905) of genes in the NT-time data set and a much larger number (10,004) of genes in the NT-weight data set (Fig. 4a; Data S5).

We then performed two comparisons: the first between DEGs in the FR data set and the NT-time data set, and the second between DEGs in the FR data set and the NT-weight data set (Fig. 4b, c). The fist comparison revealed three classes of genes associated with the time after harvest, weight loss, and dehydration rate. The most of the genes (5617) showed common temporal modulation 65 days after harvest, regardless of the final level of weight loss (22% or 30%) or the dehydration rate (Fig. 4b). However, there were 1288 NT-time-specific genes, representing the specific molecular responses to the slow NT dehydration process (65 days from harvest until 22% weight loss). Finally, there were 713 FR-specific genes representing the specific molecular responses to the rapid FR dehydration process (65 days from harvest until 30% weight loss). For each category, we focused on the top-100 DEGs ranked by |FC | (Data S6). The NT-time-specific DEGs included several members of the stilbene synthase, terpene synthase, laccase, and pectin methylesterase families, whose involvement in postharvest dehydration have been documented previously^7,10,13^. A few transcripts related to stilbene and terpene metabolism were also specifically upregulated in the FR samples, in addition to genes involved in auxin and ethylene signaling, defense (trypsin and protease inhibitor genes) and stress responses (Data S6). Among the 5617 common DEGs in the NT-time and FR samples, only 202 showed a different modulation trend (|FC | ≥ 2 between NT and FR samples at the same time point in at least one comparison) with delayed modulation under FR conditions compared with NT (Fig. 4d; Data S6). Notably, this included several genes encoding stilbene synthases, laccases, and pectin methylesterases.

A comparison of DEGs in the FR and NT-weight data set similarly revealed three classes of genes associated with different experimental variables (Fig. 4c; Data S5). However, very few DEGs (380) were specifically modulated during the FR process (65 days from harvest to 30% weight loss) but 4054 genes were specifically modulated in the NT-weight samples (120 days from harvest to 30% weight loss). These findings indicate that the natural postharvest dehydration process causes a much more profound transcriptomic reprogramming compared with the accelerated process when normalized for weight loss. Again, we focused on the top-100 DEGs ranked by |FC | , which revealed that the top-100 DEGs in the NT-weight data set showed greater modulation than the top-100 DEGs in the NT-time data set (Data S7). Moreover, we found that more than half of the genes were common to both data sets and that the genes unique to the NT-weight data set included further members of the stilbene synthase and terpene synthase families, as well as biotic and abiotic stress-response genes as described previously for Corvina berries during postharvest dehydration^7^.

The common DEGs in the NT-weight and FR samples included 5950 genes unaffected by the dehydration rate or days after harvest but influenced by the final 30% weight loss. The top-100 shared DEGs with the strongest average upregulation in both processes included 25 laccases, six stilbene synthases, four pectin methylesterases, and several genes involved in stress responses (Data S7). Interestingly, the top-100 shared DEGs with the strongest average downregulation in both processes included many markers of ripening, such as *VvMYBA* anthocyanin regulators, lipoxygenase 2 (*LOX2*), two expansin genes (*VvEXPA1* and *VvEXPA14*), and the hexose transporter gene *HT2*. Although the modulation of these genes was not specific to the NT-weight samples, our data suggest that their expression is inhibited when the postharvest dehydration process is artificially shortened (Data S7). Among the 5950 common DEGs in the NT-weight and FR samples, 1198 (20%) showed a different modulation trend (|FC | ≥ 2 between NT and FR samples at the same level of weight loss in at least one comparison). The average expression of these genes clearly showed stronger modulation in the NT-weight samples comparted to FR berries, particularly at the end of dehydration when the berries reached 30% weight loss (Fig. 4e), suggesting that the slow dehydration and long dehydration time (120 days) increase the intensity of modulation for these genes.

### Forced dehydration negatively affects the expression of quality-related gene families

**Fig. 5:**
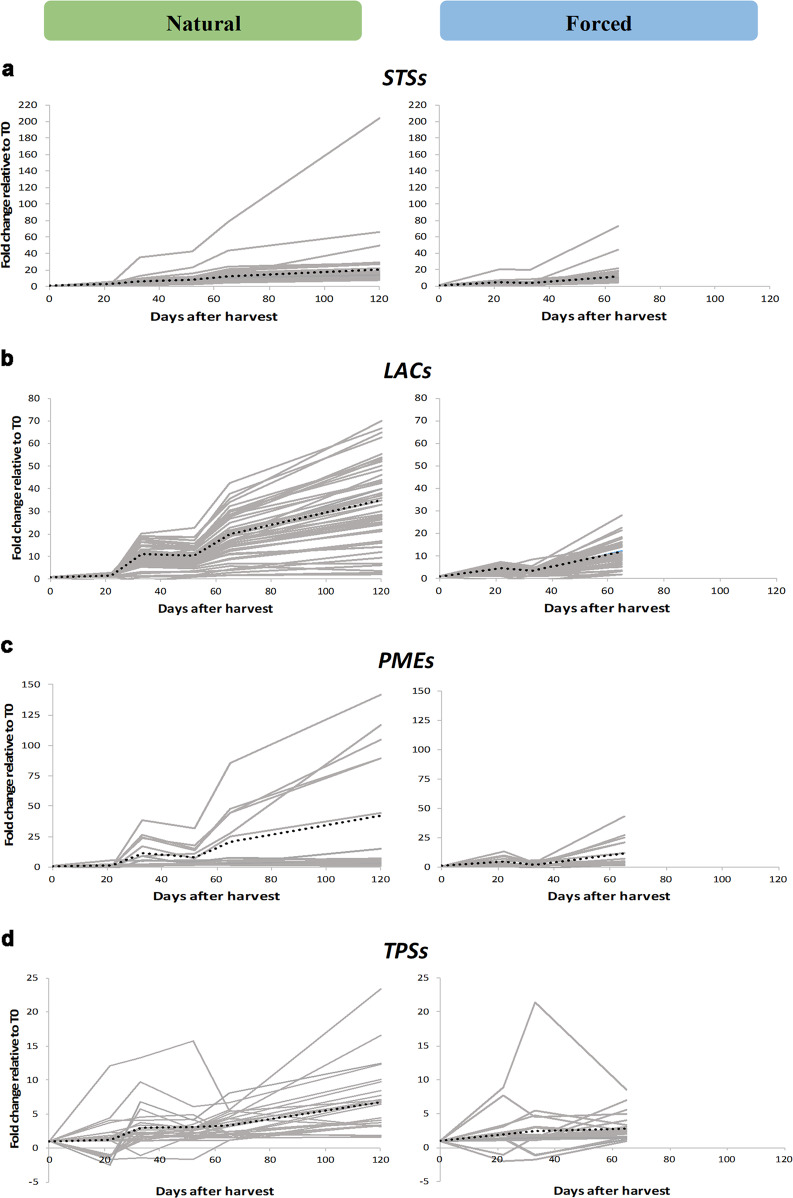
Overview of the upregulation of selected gene families during postharvest dehydration under NT and FR conditions. The trend corresponds to the fold change (FC) of each sampling time point relative to T0 during the NT (left) and FR (right) processes, for members of the: **a** stilbene synthase (*STS*), **b** laccase (*LAC*), **c** pectin methylesterase (*PME*), and **d** terpene synthase (*TPS*) gene families. Only genes differentially expressed with a |FC | ≥ 2 in at least one comparison between T0 and one of the other time points are profiled for each family (Data S5). The dotted black line represents the average trend

To study the effect of the different postharvest conditions on selected gene families, whose members may control the quality traits of dehydrated berries, we profiled the expression of laccases, stilbene synthases, pectin methylesterases, and terpene synthases under NT and FR conditions (Fig. 5). We focused on DEGs with a positive |FC | ≥ 2 in at least one comparison between T0 and one of the subsequent time points under at least one of the postharvest drying conditions (Data S5).

We observed a general steady increase in stilbene synthase gene expression under both conditions, with several genes induced strongly and rapidly but most showing a slower, more gradual increase (Fig. 5a). The final level of stilbene synthase gene expression was lower in FR than NT berries, probably reflecting the shorter lifespan of the FR berries. To verify the microarray data, we carried out confirmatory quantitative reverse transcription PCR (RT-qPCR) experiments targeting *VvSTS27* because this is among the most strongly upregulated genes in postharvest Corvina berries^7^. We conformed that *VvSTS27* mRNA is more abundant under NT than FR conditions (Figure S2a). Unlike the stilbene synthase family, most laccase genes were strongly induced during dehydration, although to a much lesser degree in FR compared with NT berries (Fig. 5b). Under NT conditions, laccase gene induction began from 22 days after harvest and there was a lag phase from 33 to 52 days, whereas the same profile was observed earlier under FR conditions. As above, we used qRT-PCR to verify the microarray data by monitoring the expression of a laccase (*VIT_18s0001g01280*) known to be strongly upregulated in postharvest Corvina berries^7^ and which shows a 40-fold modulation under NT conditions compared with only 12-fold during accelerated dehydration (Figure S2b). Overall, the higher induction of stilbene synthase and laccase genes under NT conditions is consistent with the higher stilbenoid content of the berries at the end of the NT process, suggesting that laccases may participate in the oligomerization of these compounds (Fig. 2c).

The expression profiles of pectin methylesterases under the two different conditions mirrored the trend described for laccases (Fig. 5c) and is thought to reflect their role in the rearrangement of cell wall polymers related to phenolic compound extractability during wine making^13^. As above, qRT-PCR analysis (targeting VIT_16s0022g00700) verified the expression profiles observed in the NT and FR berries (Figure S2c).

Finally, we observed two distinct expression profiles for terpene synthases under NT conditions, one featuring biphasic induction and the other featuring late-onset gene expression, overall ensuring a late surge in terpene synthase expression (Fig. 5d). Under FR conditions, the late-onset phase was missing, resulting in a less intense early wave of induction. We used qRT-PCR to profile the terpene synthase gene *VvTPS07*, which is known to be expressed strongly in postharvest Corvina berries^7^, and this confirmed that FR conditions attenuate its induction (Figure S2d). The analysis of four key gene families supported our hypothesis that slow weight loss over time encourages the onset of quality-related metabolic changes during the dehydration of berries for wine production.

**Fig. 6:**
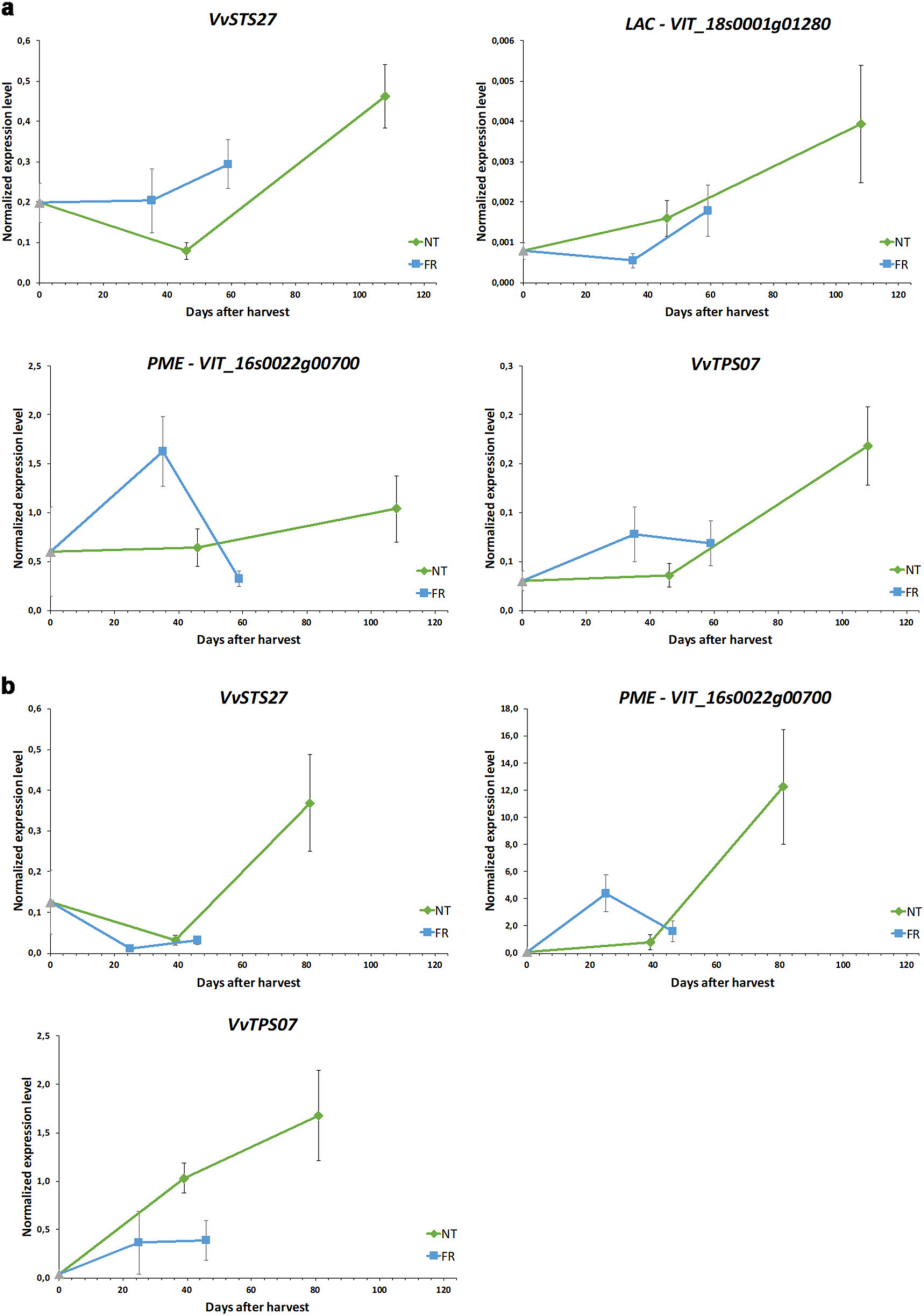
qRT-PCR analysis in cv Corvina and cv Sangiovese berries during NT and FR postharvest dehydration in 2013. **a** Expression profiles of the stilbene synthase (*STS*) gene *VvSTS27*, the laccase (*LAC*) gene *VIT_18s0001g01280*, the pectin methylesterase (*PME*) gene *VIT_16s0022g00700*, and the terpene synthase (*TPS*) gene *VvTPS07* in Corvina berries. **b** Expression profiles of the *STS* gene *VvSTS27*, the *PME* gene *VIT_16s0022g00700*, and the *TPS* gene *VvTPS07* in Sangiovese berries. Error bars represent standard deviations (*n* = 3)

To confirm the reliability of the effect of the two postharvest dehydration conditions on gene expression and to determine whether the selected genes could represent biomarkers during the natural postharvest process, we profiled their expression in Corvina and Sangiovese berries from the 2013 vintage, which were also subjected to natural and forced dehydration to reach 0%, 18%, and 30% weight loss^14^. The environmental parameters are reported in Figure S3. We monitored the expression of one member of each of the four gene families in Corvina berries and found that, in all cases, forced dehydration limited the induction of the target genes at the end of the accelerated process compared with natural dehydration (Fig. 6a). The expression profiles in the Corvina 2013 berries were also largely consistent with those of the Corvina 2011 berries reported above, with the exception of the pectin methyltransferase (Figure S2). Similar results were observed in Sangiovese berries for the same four genes (Fig. 6b) although the primers used successfully in Corvina berries did not amplify any laccase sequences in Sangiovese berries (data not shown).

## Discussion

In climacteric fruits like tomato and apple, ripening and senescence processes may occur after harvest and are associated to a marked increase in ethylene synthesis and respiration, and to metabolisms controlling changes in color, texture, aroma, and nutritional components^15^. However, ongoing metabolisms involving phytohormones, transcriptional regulation activity, and a series of physiological and biochemical changes have been described also during postharvest of non-climacteric fruits^16^. In grapevine, several studies documented the metabolic processes and the underlying molecular events that drive the berry composition changes during the postharvest phases^5,7,11,13,17^. In this work, we explored the relationship between molecular changes and dehydration kinetics in grapes placed in different environments after harvest. First, we studied the dynamics of the transcriptional program and the basis of two main gene expression profiles: (1) genes showing a steady increase or decrease in expression throughout the dehydration process, and (2) genes with a peak of induction or repression at ~18% berry weight loss. The functional description of genes belonging to these two groups provided insight into the molecular events that occur during postharvest dehydration. For example, the expression of genes related to stilbene metabolism primarily followed the profile of the first group of transcripts, with a continuous increase in expression throughout dehydration. In contrast, genes involved in cellular homeostasis and nucleic acid metabolism showed a transient spike in expression, including genes representing the PPR family of RNA-binding proteins that regulate RNA folding, splicing, degradation, cleavage, and editing^18^. The systematic induction of PPR proteins in poplar occurs in response to biotic and abiotic stress, including mechanical wounding, cold, and salinity^19^. Our data therefore suggest that dehydration stress induces transient changes in the processing of genetic information during early postharvest dehydration.

The analysis of expression dynamics for these two groups of genes revealed that the timing of expression was compressed during the FR process, such that the FR expression profiles appeared anticipated and truncated compared with those observed in the NT process. This anticipation was apparent at T1 for the first group of genes, with steadily increasing or decreasing expression, and at T2 for the second group of genes, with transient induction or repression. This indicates that the perception of osmotic stress is converted into a regulatory response earlier for the first group of genes, possibly reflecting a more immediate mechanism of regulation.

Our experimental plan allowed us to identify differences in gene expression between berries collected at the same time points but at different levels of weight loss, thus highlighting transcriptomic responses triggered by the degree of weight loss or the dehydration rate. Most DEGs (5617 genes in total) were commonly modulated under NT and FR conditions, indicating they responded similarly to duration but were not affected by differences in dehydration rate. Other DEGs were specifically modulated in the NT berries (1288) or the FR berries (713) and were therefore responsive to the long and short dehydration times, respectively. This shows that the dehydration stress caused by a weight loss above 22% in FR berries was not sufficient to trigger a full transcriptome-wide response and that the slower dehydration up to 22% weight loss in NT berries achieved major transcriptomic reprograming. One potential explanation is that basic biological functions such as gene transcription are preserved in the cells of NT berries, whereas rapid dehydration in FR berries leads to the disruption of these functions and impairs gene regulation. Indeed, the identification of several genes involved in hormone signaling and stress responses among the FR-specific DEGs reflects the higher level of osmotic stress experienced by FR cells compared with NT cells. In contrast, the most strongly modulated genes in the NT berries encoded stilbene synthases, terpene synthases, laccases, phenylalanine ammonia-lyases, and pectin methylesterases, indicating that accelerated dehydration in postharvest berries has a particular negative impact on stilbenoid, terpenoid, and cell wall metabolism.

Our experimental plan also allowed us to identify differences in gene expression between NT and FR berries with the same weight loss, sampled at different times. This revealed a huge number of DEGs specific to the NT process, indicating that a 30% weight loss must be achieved slowly in order for the berry to fully express the post-ripening transcriptomic program. Forced air ventilation therefore appears to inhibit the compositional changes that occur during natural berry dehydration. Grape genotypes that, due to their morpho-anatomical characteristics, are intrinsically prone to rapid water loss are therefore likely to show attenuated compositional changes even under natural conditions, a phenomenon that has already been reported for varieties such as Cabernet Sauvignon, Merlot, Syrah, and Oseleta^7^. One could speculate that such differences in reactivity could be at least in part associated to the rate of mesocarp cell death. Indeed, it has been reported that technical ripeness is associated with the loss of mesocarp cell vitality in Syrah berries^20^. However, in Chardonnay, Cabernet Sauvignon, and Nebbiolo berries there was no evidence of viability loss until ~40 d after veraison, and the majority (80%) of mesocarp cells remained viable past commercial harvest (26 °Brix)^21^. Fontes and collaborators^22^ confirmed that individualized cells isolated from pulp tissue of fully ripened grape berries of the white wine variety Loureiro and of the red table variety Red Globe are viable, structurally intact and physiologically active. Moreover, it was found that during development the berry water balance is a determinant of the rate at which berry cell vitality declines^23^. These studies suggest that the cell death phenomenon observed during the later stages of berry development is not generalized to the different varieties and growing conditions, and that the cell vitality is higher and, consequently, the compositional changes are more active in berries less affected by water loss.

As stated above, one of the major molecular events during natural postharvest drying is the massive upregulation of genes encoding stilbene synthases, terpene synthases, laccases, phenylalanine ammonia-lyases, and pectin methylesterases. This is a hallmark of natural berry dehydration in some genotypes and has a particularly important impact on final quality traits^5,7,13^. The induction of terpene synthases in dehydrating berries correlates with the accumulation of sesquiterpenoids, whereas the induction of pectin methylesterases correlates with skin pectin degradation^7,13^. Although we did not monitor the release of volatile organic compounds or the changing polymer composition of cell walls in this study, the gene expression profiles strongly suggest that the corresponding metabolic processes are damped under FR conditions. The sesquiterpenoid content of berries dehydrated by forced air ventilation is therefore likely to be lower than in NT berries, and the texture of the FR berry skin is likely to undergo less modification. With regard to stilbene synthases and laccases, we directly showed that the attenuated expression of both gene families in FR berries inhibited the accumulation of stilbenoids and their oligomeric derivatives.

Stilbene synthesis is highly sensitive to biotic and abiotic stress^24^. Indeed, stilbenes are among the most plastic secondary metabolites in berries of the same cultivar ripened in different years and/or different cultivation areas^25–27^. However, the increased expression of stilbene synthases is part of the late berry ripening program in some genotypes^28–30^ and low temperatures may be necessary for this metabolic process to occur. Dal Santo et al.^25^ found that stilbene synthases were strongly induced in cooler ripening seasons, and the stimulation of stilbene-related gene expression by low temperatures has been confirmed by comparing the transcriptomes of berry samples representing the distinct temperature regimes of day and night^31^, or different imposed temperature regimes after veraison^32^. The declining temperature in the late summer may therefore account for the upregulation of stilbene synthase genes during berry ripening.

We hypothesize that the strong induction of stilbene synthase genes and the accumulation of stilbenes detected during the late stages of postharvest dehydration under NT conditions could reflect the stimulatory effects of the low temperature (~6 °C) at the end of the process. However, the comparison of NT-T4 and FR-T4 samples (which experienced identical temperature conditions) revealed that the stilbenoid content of the NT berries was twice that of the FR berries, clearly showing that the slow rate of dehydration per se is needed to boost stilbenoid metabolism. The accumulation of stilbenoids at the end of the NT process may therefore reflect the combined effects of the slow dehydration rate, the longer duration of the process, and the lower temperature.

Differences in stilbene accumulation may also account for the observed differences in the state of oligomerization, with little if any oligomerization detected under FR conditions but extensive oligomerization under NT conditions. We found that the accumulation of stilbene oligomers mirrored the expression of laccases, which in turn was previously shown to match the expression of stilbene synthases^7^. Several lines of evidence indicate that plant laccases are specifically expressed in lignifying cells and are involved in the synthesis of lignin^33–37^. However, laccases catalyze the oxidation or oxidative polymerization of a broad range of phenolic compounds including proanthocyanidins^38,39^, suggesting roles in development and disease resistance that do not involve lignification^40^. We therefore speculate that the massive induction of laccases during last stages of postharvest dehydration may reflect their direct involvement in the oxidative oligomerization of stilbenes.

In conclusion, we have shown that a slow and prolonged dehydration process is needed to induce the complete panel of transcriptomic and metabolomic changes that occur in a given berry genotype after harvest. Our results show that grape dehydration kinetics should be carefully controlled by setting appropriate environmental parameters, thus supporting the transcriptomic and metabolic changes that lead to the development of desirable quality traits. By demonstrating that different dehydration regimes affect the final berry quality, we provide insight into the molecular changes that occur during postharvest dehydration and their relation to different environmental parameters.

## Material and methods

### Experimental layout

Grape bunches of *Vitis vinifera* L. cv. Corvina were harvested in 2011 and 2013 from a commercial vineyard established in 2007 at Gargagnago di Sant’Ambrogio di Valpolicella in the Masi—Serego Alighieri Estate in the Valpolicella wine district (45°31’N; 10°51’E, 165 m asl), Verona, Italy. The vineyard is located on a south-facing site with 8% maximum slope and north-south oriented rows. Vines were trained to a single-cane vertically shoot-positioned (VSP) Guyot trellis with a density of 4629 plants/ha. Grape bunches of *Vitis vinifera* L. cv. Sangiovese were harvested in 2013 from a commercial vineyard very close by the above described cv Corvina vineyard, established in 1997 at San Pietro in Cariano in the Masi—Bure Basso Estate in the Valpolicella wine district (45°31’N; 10°52’E, 145 m asl), Verona, Italy. The vineyard is located on a flat site with north east-south west oriented rows. Vines were trained to a single-cane VSP Guyot trellis with a density of 3333 plants/ha. In all cases grapes were harvested at full commercial maturity, corresponding to a level of sugars of ~21–23 °Brix, and a titratable acidity of ~5–7 g/L indicated by the enologists to make a Valpolicella-style wine. We placed ~200 kg of grape bunches on wooden racks and stored them for dehydration in Gargagnago di Sant’Ambrogio di Valpolicella, Italy (45°31′ N, 10°50′ E). In the *fruttaio*, the grapes were assigned to two dehydrating rooms where the temperature was set to reproduce the natural dehydration process^7^. The relative humidity in one room was maintained as per the natural environment (NT conditions), with the only control exerted by semi-automatic window closure. In the second room, a fan with an average air flow of ~1 m/s was placed close to the grapes, removing some of the humid air around the clusters by forced air flow (FR conditions). Weight loss was determined by periodically weighing ~15 kg (initial weight) of grapes placed on wooden boxes in the same dehydrating rooms.

Both drying processes were stopped when the berry weight loss reached ∼30%, which was after 120 days under NT conditions and 65 days under FR conditions for cv. Corvina in 2011, after 108 days under NT conditions and 59 days under FR conditions for cv. Corvina in 2013 and after 81 days under NT conditions and 46 days under FR conditions for cv. Sangiovese in 2013.

### Grape sampling and berry quality assessment

Three groups of 100 berries from each dehydrating room were randomly collected from bunches to create three biological replicates. In 2011 berries of cv. Corvina were collected at key time points (Table S1), whereas in 2013 berries of cv. Corvina and cv. Sangiovese were collected only at 0%, 18%, and 30% weight loss. For cv. Corvina berry weight loss reached ∼18% after 46 days under NT conditions and 35 days under FR conditions, for cv. Sangiovese after 39 days under NT conditions and 25 days under FR conditions.

Only healthy undamaged bunches were considered for analysis. The same biological material was used for quality assessment (total soluble solids concentration, titratable acidity, and pH) and molecular analysis (transcriptomics and metabolomics). The total soluble solids concentration, (°Brix), titratable acidity, and pH were determined as previously described^7^.

### Metabolomic analysis and data processing

Samples collected in 2011 were extracted and analyzed by HPLC-ESI-MS as previously described^26^. In brief, the extracts were analyzed by HPLC using a Beckman Coulter Gold 127 Solvent Module coupled to a Bruker Esquire 6000 ion trap mass spectrometer (Bruker Daltonik GmbH, Bremen, Germany) equipped with an electrospray ionization source. Negative ion spectra were recorded in the range 50–2000 m/z (full scan mode, 13,000 m/z per second). For metabolite identification, MS/MS and MS3 spectra were recorded in negative mode. Metabolites were putatively identified by comparing the m/z values, fragmentation patterns (MS/MS and MS3) and retention times of each signal with those of an in-house library of authentic standards. When commercial standards were not available, m/z and fragmentation patterns were compared with those published in the literature or on-line databases such as MassBank (www.massbank.jp/en/database.html) and Human Metabolome Database (http://www.hmdb.ca/).

### RNA extraction

Total RNA was extracted from ~200 mg of ground berry tissue (pericarp without seeds) using the Spectrum Plant Total RNA kit (Sigma-Aldrich) with some modifications^6^. RNA quality and quantity were determined using a Nanodrop 2000 spectrophotometer (Thermo Fisher Scientific) and a Bioanalyser Chip RNA 7500 series II (Agilent Technologies).

### Microarray analysis and data processing

We hybridized 10 µg of total RNA per sample to a 090818_Vitus_exp_HX12 microarray chip (Roche/NimbleGen) representing 29,549 predicted grapevine genes (http://ddlab.sci.univr.it/FunctionalGenomics/) followed by scanning and image analysis as previously described^6^. The reported values (figures and data sets) are means of three biological replicates, and Pearson’s correlation analysis was therefore applied to evaluate the robustness of the replicates. PCA in SIMCA-P v13.0 was applied to the entire data matrix. The loadings of the first and second principal components were ordered, and the first and last 100 genes were extracted to investigate their functional categories.

Differentially expressed genes were identified by applying a *t* test using TMeV v4.8 (http://www.tm4.org/mev) with a *P* value of 0.01%, followed by multiclass significance analysis of microarrays (SAM) with a false discovery rate (FDR) of 0.01% to extract genes that were significantly modulated during forced dehydration. To determine the effect of timing and weight loss, SAM was applied to two subsets of the microarray data. The first subset (NT-time data set) included NT T0, T1, T2, and T4, allowing a time-based comparison with the forced dehydration process, and the second subset (NT-weight data set) included NT T0, T2, T3, and T5, allowing a weight loss-based comparison between the processes. The list of genes with the highest level of modulation in each category was determined by ranking the mean value of the fold-changes at each time point of the data set(s) relative to T0.

### Reverse transcriptase qRT-PCR

For the 2011 berry samples, qRT-PCR was carried out using the same RNA samples used for the microarray analysis. For the 2013 berry samples, total RNA was extracted from ground pericarps and DNA was removed by incubation with RQ1 RNase-Free DNase (Promega). The cDNA synthesis and qRT-PCR analysis were carried out as previously described^29^. Each expression value was measured in triplicate and normalized to the internal control *VvUBIQUITIN1*. The primer sets are listed in Table S3. Amplification efficiency was calculated using LingRegPCR^41^ and standard error values were calculated according to Pfaffl et al.^42^.

### Accession numbers

Grape berry microarray expression data are available in the Gene Expression Omnibus under the series entry GSE138021.

## Supplementary information

Supplemental Material

data set S1

data set S2

data set S3

data set S4

data set S5

data set S6

data set S7
