# Supplementary material for: Rapid dehydration of grape berries dampens the post-ripening transcriptomic program and the metabolite profile evolution
Source: Hortic Res. 2020 Sep 1;7:141. doi: 10.1038/s41438-020-00362-5 (PMC7459318; doi:10.1038/s41438-020-00362-5)

Table S1 Sample collection details. During the NT process, berries were collected at six time points (T0, T1, T2, T3, T4 and T5). During the FR process, berries were collected at four time points (T0, T1, T2, and T4). Under both conditions, T0 corresponds to fully-ripe berries, T1 to 22 days after harvest, T2 to 33 days after harvest, and T4 to 65 days after harvest. Furthermore, in terms of percentage weight loss, NT-T2 corresponds to FR-T1 (14%), NT-T3 to FR-T2 (18%), and NT-T5 to FR-T4 (30%).

	**Sampling Time Points**					
	**T0 (Harvest)**	**T1**	**T2**	**T3**	**T4**	**T5**
Sampling Data	September 12th 2011	October 4th 2011	October 14th 2011	November 3rd 2011	November 16th 2011	January 10th 2012
**NT Weight loss (%)**	0	10	14	18	22	30
**NT Dehydration day**	0	22	33	52	65	120
**FR Weight loss (%)**		14	18		30	
**FR Dehydration day**		22	33		65	

Table S2 Titratable acidity and pH values during postharvest dehydration. (n = 3 ± standard deviation).

Time point	Process	Titratable acidity (g/L of tartaric acid)	pH
**T0**	NT/FR	5.10 ± 0.11	3.18 ± 0.06
**T1**	NT	5.59 ± 0.11	3.51 ± 0.04
	FR	5.46 ± 0.26	3.43 ± 0.12
**T2**	NT	5.48 ± 0.17	3.46 ± 0.05
	FR	5.44 ± 0.26	3.44 ± 0.08
**T3**	NT	5.45 ± 0.15	3.44 ± 0.05
**T4**	NT	4.51 ± 0.14	3.49 ± 0.04
	FR	4.64 ± 0.31	3.59 ± 0.10
**T5**	NT	5.17 ± 0.16	3.75 ± 0.07

TABLE S3 Primer sets.

GENE	GENE ID	SEQUENCE 5'→3'
VvTPS07	VIT_18s0001g04280	Fw - TACCTTAGCCGCCCCTAATG<br>Rev - AGCACTACCAAACCGAGAGA
VvSTS27	VIT_16S0100G00990	Fw - ATTCTGCAGGTGCCATTGCA<br>Rev - ATACCAAGTGGGTCAAAAGCC
Laccase	VIT_18s0001g01280	Fw - TCACAGTGATTGGACCCGAA<br>Rev - AATCAGAGGCATTGGGGTCA
Pectinesterase family	VIT_16s0022g00700	Fw - AGTGTCGCCAACTTTATAGCT<br>Rev - ATCATCATAGACCAGACCTGA
WRKY DNA-binding protein 72	VIT_17s0000g05810	Fw - CTTACATCAATAATGGGTGCTA<br>Rev - TGACTGATAGGTAGAAACAGC
Alpha-dioxygenase	VIT_14s0066g01670	Fw - GGATCGTCATTACCCAGAGA<br>Rev - GATCAATGGGGAACACGAAG
VvUBIQUITIN1	VIT_16s0098g01190	Fw - TCTGAGGCTTCGTGGTGGTA<br>Rev - AGGCGTGCATAACATTTGCG

FIGURE S1 Principal component analysis of transcriptomic data from Corvina berries collected during postharvest dehydration in 2010.

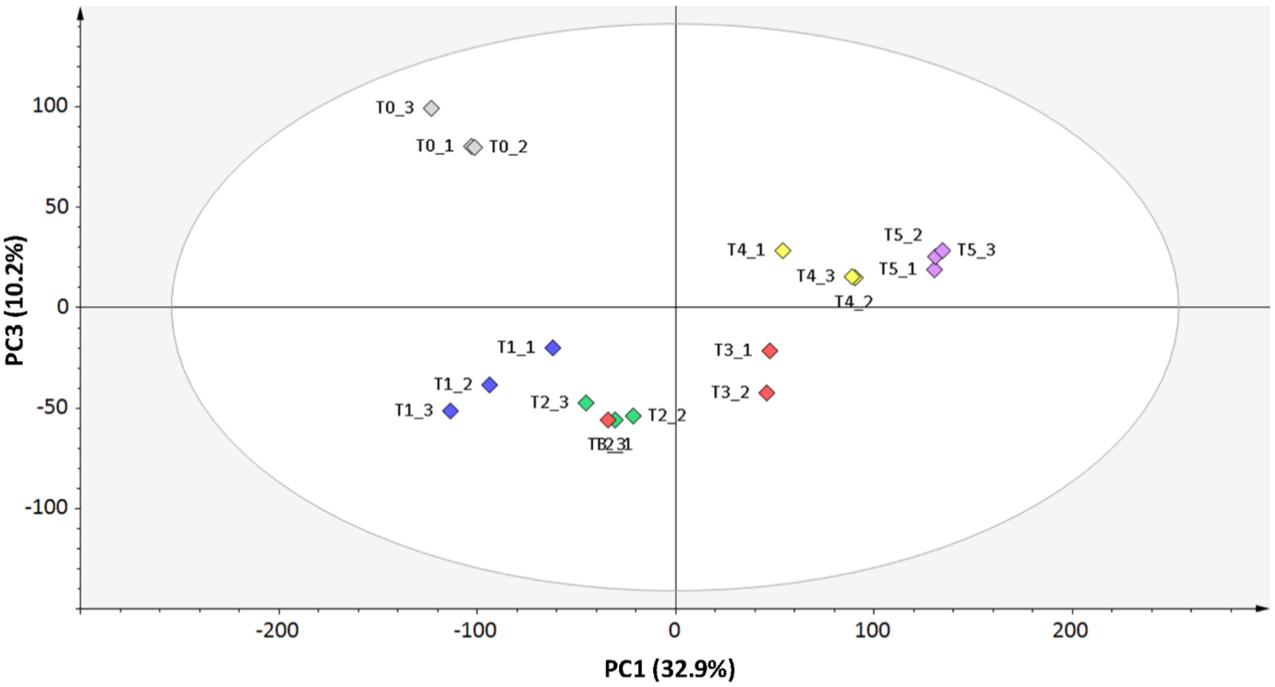

FIGURE S2 qRT-PCR verification of microarray data. Expression profiles obtained by microarray analysis (left) and qPCR (right) for (a) *VvSTS27* (b) *LAC* (c) *PME*, and (d) *VvTPS07* under NT and FR postharvest dehydration conditions. Microarray data are reported as the average of three biological replicates. Error bars represent standard deviations (n = 3).

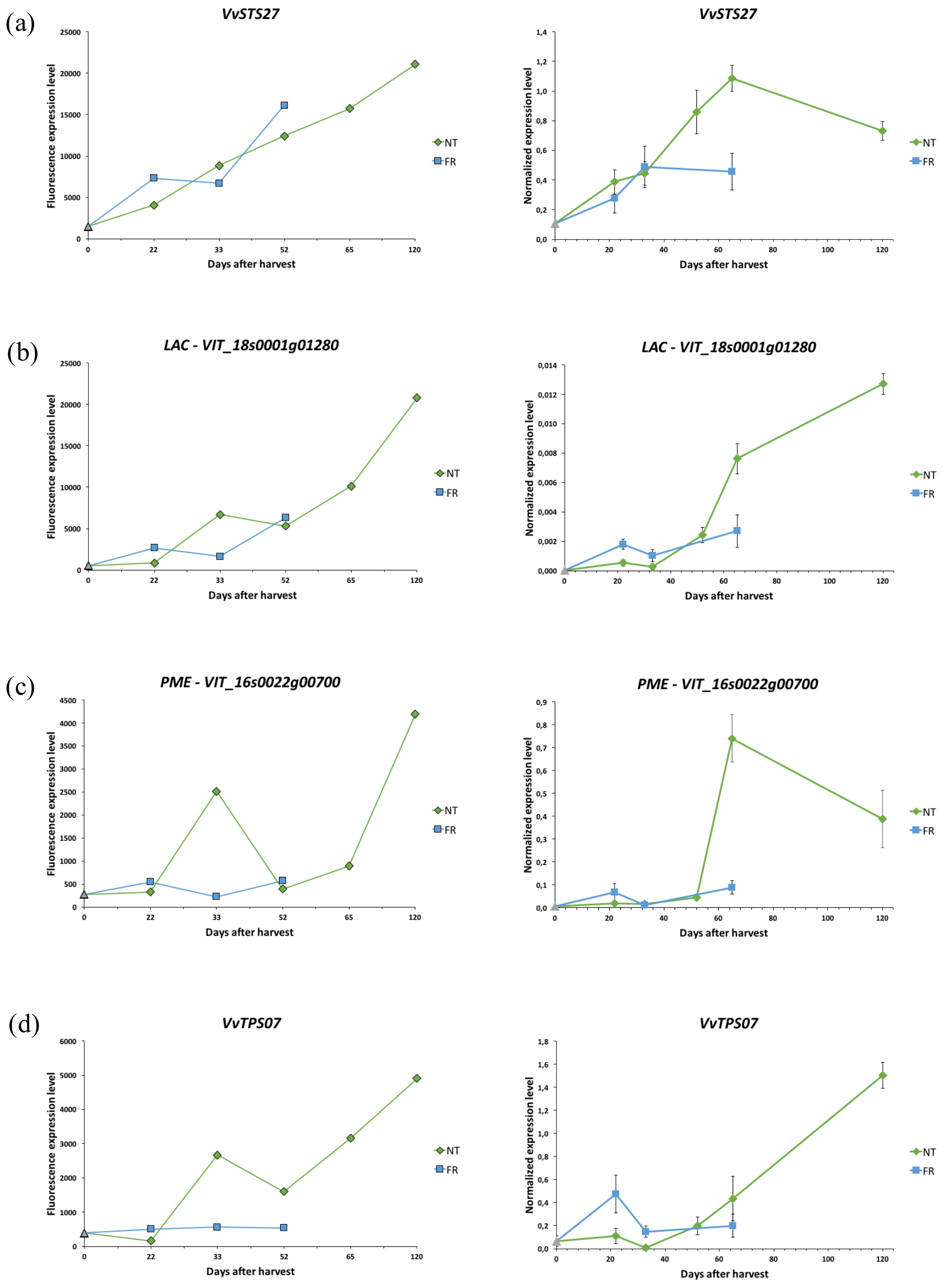

FIGURE S3 Overview of the NT and FR postharvest dehydration processes during the 2013 vintage. (a) Dehydration kinetics of Corvina and Sangiovese berries under NT and FR postharvest conditions, with sampling time points highlighted. For both varieties and under both conditions, T0 corresponds to fully-ripe berries, T1 to berries sampled at 18% weight loss and T2 to berries sampled at 30% weight loss. (b) Trends of relative humidity in the two dehydrating rooms used to reproduce NT and FR dehydration processes. (c) Trends of temperature in the two dehydrating rooms.

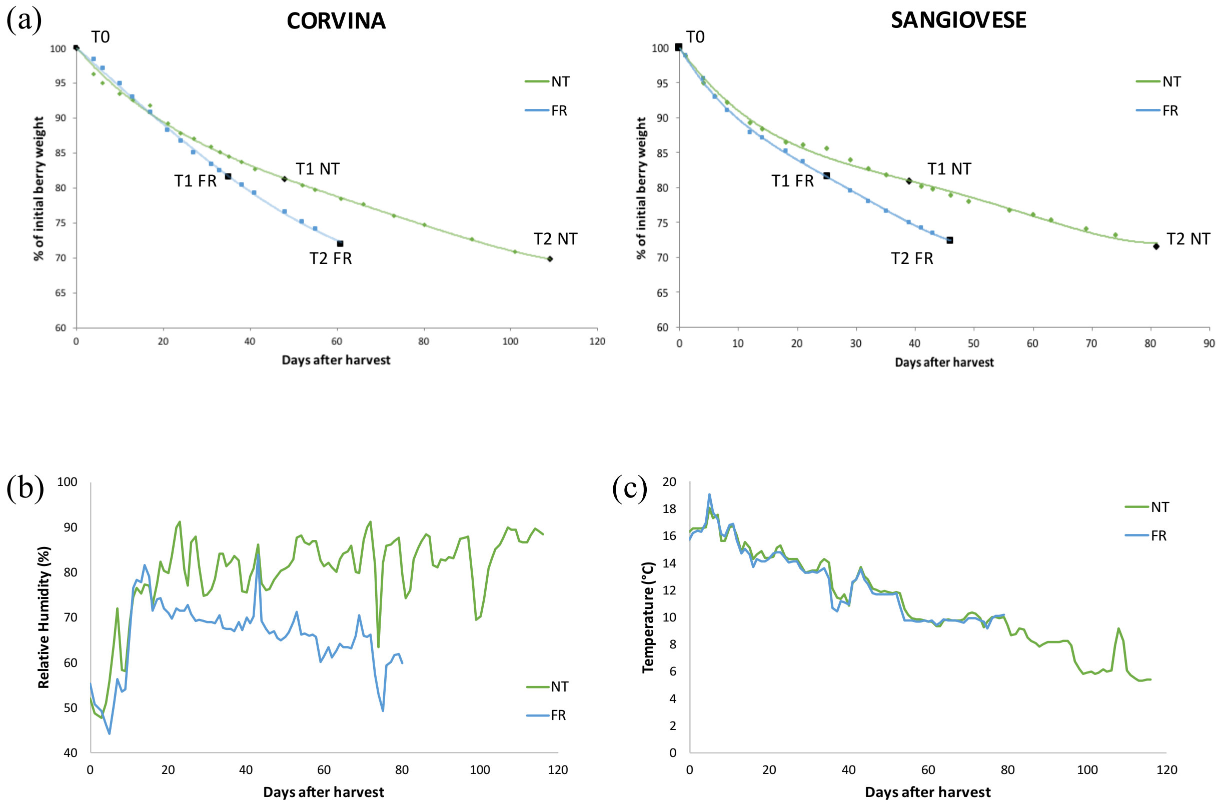

Supplement: Supplementary file 1 — Supplemental Material [file 41438_2020_362_MOESM1_ESM.pdf]
